# Vital Role of Visceral Adipose Tissue in Maintaining Cognitive Functions

**DOI:** 10.3390/ijms26146597

**Published:** 2025-07-09

**Authors:** Rina Shirafuji, Yoko Amagase, Ai Goto, Yoshinori Takei

**Affiliations:** 1 Faculty of Medicine, Toho University, 5-21-16 Omori-nishi, Ota-ku, Tokyo 143-8540, Japan; 2 Faculty of Pharmacy, Osaka Medical and Pharmaceutical University, 4-20-1 Nasahara, Takatsuki 569-1094, Osaka, Japan; 3 Department of Pharmacology, Faculty of Medicine, Toho University, 5-21-16 Omori-nishi, Ota-ku, Tokyo 143-8540, Japan; 4 Department of Translational Research & Cellular Therapeutics, Faculty of Medicine, Toho University, 5-21-16 Omori-nishi, Ota-ku, Tokyo 143-8540, Japan; 5 YOKOYAMA Kazuya Cancer Research Institute, 2F Ueno Yokoyama Bidg, 1-4-8 Ueno, Taito-ku, Tokyo 110-0005, Japan

**Keywords:** aging, obesity, exercise, fasting, BDNF, cognitive function, hippocampus, visceral adipose tissue

## Abstract

The aging process involves a decline in certain cognitive abilities. Cognitive aging progresses more quickly with obesity and more slowly with exercise and fasting. All of these conditions have strong impacts on white adipose tissue, which suggests that this tissue may play a pivotal role in the progression of cognitive aging. Brain-derived neurotrophic factor (BDNF), a neurotrophin indispensable for maintaining brain functions, becomes insufficient with age. Obesity also decreases the BDNF level in the hippocampus. This deficiency not only results in cognitive impairment but increases susceptibility to obesity. Both exercise and fasting increase the BDNF level in the hippocampus. Our study demonstrates that the chemokine ligand CX3CL1 in white adipose tissue is involved in the regulation of the BDNF level in the hippocampus. Aging reduces CX3CL1 expression, interfering with the mechanisms. Other studies have suggested that obesity increases adipose CX3CL1 expression; however, CX3CL1 augmented under obese condition may not contribute to the promotion of the BDNF level in the hippocampus. This suggests that the malfunction of the adipose CX3CL1-mediated mechanism could be involved in the downregulation of the hippocampus BDNF level under obese conditions. Studies have also suggested that the adipose CX3CL1-mediated mechanism appears to be involved in the exercise-induced promotion of BDNF expression in the hippocampus. Its involvement in the fasting-induced BDNF promotion is still unknown. Therefore, aging, obesity, and exercise appear to affect white adipose tissue to regulate the hippocampus BDNF level. While further studies are required to elucidate the precise role of the adipose CX3CL1-mediated regulation of BDNF expression, studies on white adipose tissue may provide new therapeutic targets for preventing age-associated cognitive decline.

## 1. Introduction

Aging is characterized by progressive physiological decline with increasing chronological age [1]. The aging process includes complex interactions within and between organs, and it remains poorly understood. The number of older adults is increasing, leading to an increased burden on health services and society [2,3].

Adipose tissue consists of brown, beige, and white adipose tissue, and plays a crucial role in modulating whole-body energy metabolism [4]. Brown adipose tissue consumes chemical energy to produce heat, regulating body temperature [5,6]. Beige adipocytes are scattered within white adipose tissue and can be induced to switch from a white-adipocyte-like phenotype to a brown-adipocyte-like phenotype [7]. White adipose tissue is mainly composed of unilocular adipocytes containing large lipid droplets [8]. Adipocytes incorporate free fatty acids (FFAs) and glucose from blood plasma, and convert them into triglycerides to prepare for shortages of energy in the future [9,10]. Moreover, white adipose tissue secretes hormones, such as adiponectin, leptin, and resistin, and cytokines such as tumor necrosis factor-alpha (TNF-α), IL-6, and monocyte chemotactic protein-1, which regulate glucose metabolism, insulin sensitivity, tissue repair, and low-grade systemic inflammation related to obesity [11,12,13,14,15].

White adipose tissue appears to be among the most vulnerable tissues during aging, and it undergoes substantial changes in mass, distribution, cellular composition, secretory profiles, and insulin responsiveness during aging [16]. These changes not only have strong impacts on various organs, including adipose tissue itself, exerting multiorgan dysfunction and disability, but correlate with metabolic dysfunction and chronic low-grade systemic inflammation [17].

Obesity is defined as the excessive deposition of fat in white adipose tissue. Exercise and fasting consume triglycerides in white adipose tissue, shrinking the tissue mass. Thus, all of these conditions have strong impacts on white adipose tissue. While obesity appears to accelerate the aging process of cognitive functions, both exercise and fasting slow or reverse it. These findings may suggest a close relationship between the status of white adipose tissue and cognitive aging.

In this narrative review, we will firstly provide a brief summary of the current knowledge regarding the impacts of aging, obesity, exercise, and fasting on white adipose tissue and cognitive functions. Secondly, we will introduce the new connection between white adipose tissue and the regulation of brain-derived neurotrophic factor (BDNF) in the hippocampus. Thirdly, we will discuss how aging, obesity, exercise, and fasting influence this new relationship in regulating cognitive functions. There are existing excellent reviews on the relationship between the factors secreted from white adipose tissue and aging [4,18] and between the intestinal environment and aging [19,20], so these topics are not mentioned in this review.

## 2. Effects of Aging on White Adipose Tissue

White adipose tissue is divided into two biologically distinct groups, subcutaneous and visceral adipose tissues. Subcutaneous adipose tissue is typically considered beneficial for metabolism, whereas visceral adipose tissue is often considered to be harmful [21]. While obesity is an important risk factor for many diseases, visceral adipose tissue is strongly related to many health conditions in humans [22]. Many studies have demonstrated that fat accumulation in visceral adipose tissue is a risk factor for insulin resistance [23], type 2 diabetes mellitus [23], cardiovascular disease [24], stroke [25], heart failure [26], cognitive decline [27], and Alzheimer’s disease [28]. The surgical removal of visceral fat from young moderately obese rats reduced insulin levels, improved glucose tolerance, and lowered pro-inflammatory cytokines in serum [29]. Visceral fat removal from a mouse model of diet-induced obesity and type 2 diabetes mellitus restored the metabolic parameters and serum cytokine levels, and it attenuated the impairment of insulin signaling [30]. These findings demonstrate the major role of visceral adipose tissue in the adverse health effects of general obesity.

By contrast, in a study consisting of 15 participants with obesity who had a similar BMI, several metabolic parameters were assessed before and 10–12 weeks after having approximately 10.5 kg of subcutaneous abdominal fat removal [31]. Subcutaneous fat removal did not alter insulin action in the liver, muscle, and adipose tissue, nor did it alter the blood concentrations of C-reactive protein, interleukin-6, tumor necrosis factor-α, adiponectin, glucose, insulin, and blood lipids [31]. This study suggests that subcutaneous fat accumulation contributes only minimally to the adverse health effects of obesity.

Fat mass is commonly increased in older adults [32]. The excess fat is preferentially stored in visceral adipose tissue, and the mass of subcutaneous adipose tissue decreases [33]. The age-associated changes in fat distribution are correlated with an increased risk of metabolic abnormalities, particularly insulin resistance accompanied with an increased risk of cardiovascular disease and diabetes [32,34]. The underlying mechanisms of age-associated changes in fat distribution are not fully understood. The changes in older adults could enhance the adverse health effects of visceral adipose tissue.

## 3. Obesity Accelerates the Aging of White Adipose Tissue

There are several common features between aging and obesity, and adults with obesity are thought to be prematurely aged individuals [35]. Both aging and obesity are associated with systemic inflammation [36,37,38], oxidative stress [39,40], and changes in microbiota composition [41,42]. Thus, obesity appears to be a state of accelerated biological age [43].

Cellular senescence is the cellular stress response triggered by the proteins working in cell cycle arrest, such as p16, p53, and p21. Senescent cells stop cell proliferation, resist apoptosis, and secrete a variety of signaling molecules. The secretion of signaling molecules from senescent cells is called the senescence-associated secretary phenotype (SASP), which contributes to the promotion of chronic inflammation. Recent studies have indicated that the number of senescent cells increases in most organs and tissues with age, which in turn reduces the number of functional cells [44]. The killing of senescent cells by selective apoptosis improves age-associated decline in tissue functions, extends lifespan, and delays the onset of age-related illnesses [45]. In the early stage of aging, senescent cells accumulate in white adipose tissue, affecting adipose cell differentiation and protein secretion [46]. Senescent cells are also accumulated in the adipose tissue with obesity, which is related to diabetes [47].

Reactive oxygen species (ROS) are produced in senescent cells and obese adipose cells, and they damage telomeres and induce cell death [48,49]. Individuals with overweight or obesity have shorter telomeres, which is also observed in older adults [50]. ROS activate the transcription factor nuclear factor κB, inducing the secretion of pro-inflammatory cytokines and the expression of the genes that regulate apoptosis and cell senescence [51,52]. Moreover, ROS helps in the development of cellular senescence through the activation of the p53 gene [53]. These findings suggest that obesity accelerates the aging of white adipose tissue.

## 4. Obesity Accelerates Brain Aging

Overweight and obese adults in midlife have an increased risk of dementia in later life stages [54,55,56]. A study including 527 cognitively healthy subjects (female and male) with an age range of 20–87 years old demonstrated that the white matter volume decreases in an age-associated manner, which is associated with a greater degree of atrophy [57]. Obesity/overweight accelerates age-associated volume reduction [57]. The white matter volume of 50-year-old obese/overweight subjects is similar to that of 60-year-old lean subjects [57]. Obesity is increasingly recognized as an important hallmark of the aging process.

## 5. BDNF Has a Critical Role in Maintaining Brain Functions

BDNF is a neurotrophin, promoting the viability and functional integrity of certain neurons. Its expression begins in the early stages of development and persists through adult life [58]. *Bdnf-/-* mice exhibit gross neurodevelopmental and sensory defects and are homozygous lethal [59]. In mice with heterozygous deletion of the *Bdnf* gene, spatial learning is impaired [60], and the proliferation of neural stem cells (NSCs) in the hippocampus is reduced [61]. Moreover, the death of new-born neurons is more frequently observed in adult mice with the heterozygous deletion of the *Bdnf* gene [61]. The hippocampus-specific knockout of the *Bdnf* gene decreases adult neurogenesis and impairs novel object recognition and spatial learning [62]. Conversely, the stimulation of BDNF expression increases adult neurogenesis in the hippocampus [63]. Increased BDNF expression augments in vivo proliferation, differentiation, axonal path migration, and the maturation of NSCs in the hippocampus [64,65].

BDNF expression is downregulated in the brains of older adults with decreased learning ability [66,67]. Decreased BDNF expression results in impaired memory, neurodegeneration, and other cognitive impairments in older adults [68]. BDNF expression is also decreased in the brains of patients with major depressive disorder [69]. Multiple factors have been reported to be involved in the age-related decline of the BDNF level in the hippocampus, which includes changes in gut microbiota [70], life style [71], stress responses [72,73], and astrocyte phenotypes [74]. Recently, we found that age-associated alterations in visceral adipose tissue contributed to the regulation of the hippocampus BDNF levels [75]. The mechanisms underlying the decrease in the level of BDNF expression in older adults are still not fully understood.

## 6. Inverse Relationship Between Obesity and BDNF

The BDNF gene has been associated with obesity in genome-wide association studies [76]. Intracerebroventricular injection of BDNF reduces food intake, limits weight gain, and enhances locomotion [77,78]. These findings suggest that BDNF is involved in the regulation of food consumption.

Consuming a high-sugar or high-fat diet decreases the BDNF level in the rat brain [79,80,81]. High-fat diet consumption reduces BDNF expression, impairing synaptic plasticity in the hippocampus, consequently causing a decrease in cognitive functions [82,83,84,85]. Moreover, a high-fat diet increases brain oxidative stress, stimulating neuroinflammation and decreasing BDNF levels [86,87]. Therefore, obesity appears to decrease the BDNF level in the brain, which is consistent with the finding that obesity accelerates brain aging, as discussed in the previous section. These reports suggest that obesity promotes cognitive aging via the dysregulation of BDNF, and reciprocally, the age-associated decline in BDNF levels may promote not only cognitive aging but vulnerability to obesity.

## 7. Exercise Increases the Hippocampus BDNF Expression

Physical activity, including exercise training, reduces the white adipose tissue mass and influences the key drivers of aging, including chronic inflammation, mitochondrial dysfunction, myokine release, autophagy, oxidative damage, and insulin-like growth factor signaling [88,89,90]. Exercise improves physical functions, reducing the burden of chronic diseases and overall premature mortality, including cause-specific mortality from cardiovascular disease, cancer, and chronic lower respiratory tract diseases [91,92,93].

Exercise is beneficial for cognitive functions [94,95], with the effect being most prominent in older adults [96]. Moreover, exercise ameliorates the symptoms of neurological disorders, such as depression, epilepsy, stroke, Alzheimer’s disease, and Parkinson’s disease [97,98,99,100,101]. Exercise not only increases synapse plasticity, adult hippocampus neurogenesis, the size of the hippocampus, and blood flow to the hippocampus but it changes the morphology of dendrites and dendritic spines [94,95,102].

In animal models, moderate exercise induced BDNF expression in various regions of the brain, most robustly in the hippocampus [94]. In mice, voluntary exercise for 2–3 days increased BDNF expression in the dentate gyrus [103]. The exercise-induced augmentation of BDNF levels was maintained throughout several weeks [100]. Blocking BDNF signaling inhibited the exercise-induced improvement in spatial memory and synaptic protein expression [104,105]. While controversial results have been observed in young animals [106], BDNF signaling appears to be associated with the beneficial effects of exercise, at least for older animals. The precise mechanism by which exercise induces BDNF expression remains to be elucidated. It is still unclear how much exercise is necessary to maintain cognitive functions, or how long the effects of exercise on cognitive functions last.

## 8. Fasting Augments the Hippocampus BDNF Expression

In addition to exercise, fasting is recognized to be another intervention that improves the process of aging and reduces the adipose tissue mass [107]. Fasting is a method of stressing the body and causing metabolic changes by restricting food intake [108], resulting in various benefits for health, including weight management, insulin resistance, reduced inflammation, and improved cognitive functions [109,110]. Intermittent fasting, which restricts eating in specific periods during the day or week, has been recognized as an alternative to continuous fasting. It reduces visceral fat volume and improves insulin resistance, dyslipidemia, and inflammatory cytokines [111,112,113,114,115,116]. In addition to intermittent fasting, fasting-mimicking diets that allow for some food intake without time restriction can also promote healthy aging [117].

During fasting, the body consumes the glycogen and fat stored in the body as alternative energy sources [118], whereby weight loss and a reduced mass of white adipose tissue can be expected [119]. Fasting for 10 to 14 h or more triggers a series of metabolic responses, including the digestion of liver glycogen, the breakdown of triglycerides into free fatty acids in adipocytes, and the release of free fatty acids into the circulation [120]. Circulating free fatty acids are transported to hepatocytes and are converted into ketone bodies, such as acetoacetate and β-hydroxybutyrate. Ketone bodies are metabolized into acetyl CoA, entering the tricarboxylic acid cycle to generate ATP. β-Hydroxybutyrate also serves as a signaling molecule, activating transcription factors, like the cyclic AMP response element binding protein and nuclear factor κB, and promoting BDNF expression in neurons.

Although increasing BDNF levels through fasting should improve cognitive functions in aged animals, the effects of fasting on the aging of cognition/memory formation functions are not conclusive. Some papers have reported improvements in cognitive aging [110,121,122], but other studies have found limited or no improvement [123,124]. Moreover, the effect of β-hydroxybutyrate on BDNF expression is not fully confirmed in humans. Ketosis achieved via a ketogenic diet and/or ketogenic supplements does not increase blood BDNF levels in humans [125]. The plasma levels of BDNF are primarily derived from efflux from the brain to circulation [126,127]. It appears that circulating ketone bodies may not always promote BDNF. While fasting induces BDNF expression, the mechanisms by which fasting promotes brain BDNF expression are not fully understood.

## 9. Interim Conclusion

Briefly, obesity/overweight can accelerate the aging of white adipose tissue. Exercise and fasting help to shrink the mass of visceral white adipose tissue. Cognitive aging is closely related to the BDNF level in the hippocampus. While aging and obesity decrease the BDNF level, exercise and fasting increase it. The mechanism connecting the status of visceral adipose tissue and the hippocampus BDNF level is not fully understood. In the next section, we introduce a new concept for the contribution of white adipose tissue to the regulation of the hippocampus BDNF level.

## 10. Young Visceral Adipose Tissue Upregulates the Hippocampus BDNF Protein Level

**Figure 1:**
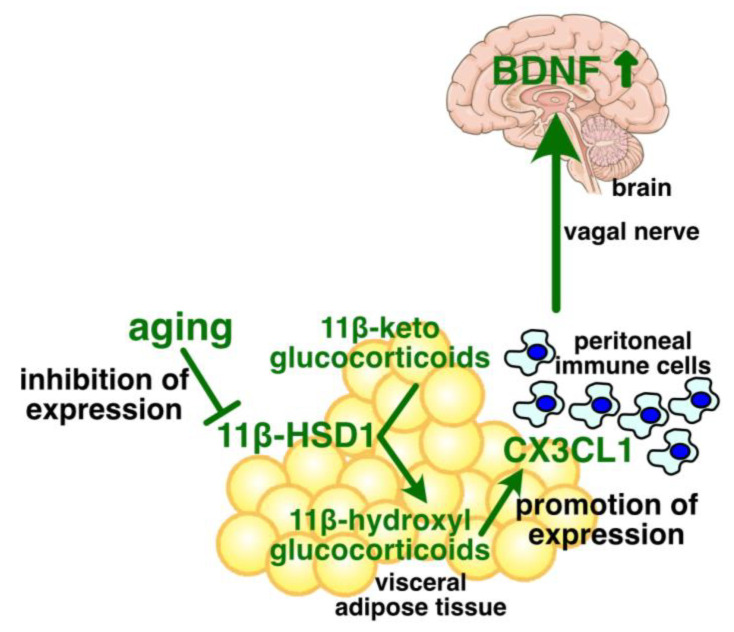
Novel relationship between visceral adipose tissue and the brain, and its attenuation with advancing age. The glucocorticoid activating enzyme 11β-HSD1 maintains glucocorticoid availability in the visceral adipose tissue to promote CX3CL1 expression [51]. The adipose CX3CL1 increases the protein levels of BDNF in the hippocampus via modulating the peritoneal macrophages and the vagal nerve [78]. The expression of adipose 11β-HSD1 is decreased with advancing age, which results in diminished protein levels of adipose CX3CL1 and the hippocampus BDNF, and consequently induces the decline in cognitive functions. The details are described in the main text.

Although visceral fat accumulation appears to be responsible for the adverse effects of general obesity, as mentioned in Section 2, our study demonstrates that visceral adipose tissue is involved in promoting the BDNF protein level in the hippocampus, at least in young individuals and those with non-obese conditions [75]. Both the BDNF protein level in the hippocampus and the chemokine ligand CX3CL1 level in visceral adipose tissue were reduced in aged mice under non-obese conditions. The visceral adipose tissue-specific knockdown of CX3CL1 reduced the hippocampus BDNF levels in young mice. Conversely, a single intraperitoneal injection of CX3CL1 recovered the hippocampus BDNF levels in aged mice. These results suggest a novel inter-tissue crosstalk involved in the regulation of the hippocampus BDNF levels (Figure 1).

When CX3CL1 was administered into the peritoneal cavity of aged mice for 2 weeks, to compensate for the reduced CX3CL1 expression in visceral adipose tissue, the hippocampus BDNF level was increased and age-related impairment of cognitive functions was improved in male and female mice [128]. This suggests that the age-associated decline in CX3CL1 expression in visceral adipose tissue is sufficient to induce cognitive impairment in aged mice. The transplantation of peritoneal cells from CX3CL1-treated aged mice into the peritoneal cavity of normal aged mice improved memory regarding novel object recognition in the recipient aged mice [128]. Moreover, vagotomy inhibited the CX3CL1-induced promotion of the hippocampus BDNF expression [128]. These results suggest that peritoneal CX3CL1 promotes BDNF in the hippocampus primarily through modulating the peritoneal immune cells and the vagal nerve (Figure 1). Peritoneal CX3CL1 may not need to enter the circulation to increase the BDNF protein level, and appears to contribute prominently to the regulation of the hippocampus BDNF levels. Visceral adipose tissue is in the peritoneal cavity, and its CX3CL1 expression is the highest in the peritoneal tissues, according to “The Human Protein Atlas” (https://www.proteinatlas.org/ENSG00000006210-CX3CL1/tissue, accessed on 6 July 2025) [129].

## 11. Age-Associated Regulation of Adipose CX3CL1 Expression

The glucocorticoid-activating enzyme, 11β hydroxysteroid dehydrogenase type 1 (11β-HSD1) is expressed in the target tissues of glucocorticoids, such as the liver and adipose tissue [130]. This enzyme converts hormonally inactive 11β-keto glucocorticoids to active 11β-hydroxylated forms [130]. In short, the enzyme converts hormonally inactive dehydrocorticosterone and cortisone to corticosterone and hydrocortisone (cortisol), respectively [130]. Mice deficient in 11β-HSD1 are protected from excessive glucocorticoid-induced glucose intolerance, hyperinsulinemia, hepatic steatosis, adiposity, hypertension, myopathy, and dermal atrophy [131]. This demonstrates that 11β-HSD1 is essential for glucocorticoids to exert proper effects on their target tissues.

Both the protein level and enzymatic activity of 11β-HSD1 were reported to be lower in visceral adipose tissue of aged mice compared to young mice [75,131]. Visceral adipose tissue-specific knockdown of 11β-HSD1 expression decreased both the adipose CX3CL1 expression and the hippocampus BDNF levels in young mice [75]. Thus, the age-associated decline in CX3CL1 expression in visceral adipose tissue appears to be caused by the reduced expression of 11β-HSD1 in the tissue. This may suggest that glucocorticoids are involved in the regulation of the adipose CX3CL1 expression, considering that 11β-HSD1 regulates glucocorticoid availability in visceral adipose tissue [131,132]. An in vitro study indicated that naturally occurring glucocorticoids, such as corticosterone and hydrocortisone, increased CX3CL1 expression in a concentration-dependent manner [75]. Therefore, circulating glucocorticoids may contribute to maintaining the hippocampus BDNF levels through the induction of CX3CL1 expression in visceral adipose tissue in young mice, but not in aged mice (Figure 1).

The mechanism underlying the age-associated reduction in 11β-HSD1 expression in visceral adipose tissue is still unknown; however, it is speculated that this reduction is related to the age-associated regulation of DNA methylation in the promoter region of the *HSD11B1* gene. The methylation of the *HSD11B1* promoter has been reported to have a role in the placenta [133], skeletal muscle [134], and adipose tissue [135]. DNA methylation is well known to change during aging [136]. Various recent studies have demonstrated the presence of age-related CpG sites (AR-CpGs), which are either hypermethylated or hypomethylated [137,138,139,140,141]. Thus, it is a reasonable speculation that age-associated changes in DNA methylation may affect the expression levels of 11β-HSD1 in white adipose tissue.

## 12. Effects of Obesity on Glucocorticoids and 11β-HSD1 in Adipose Tissue

Patients with abdominal obesity have elevated cortisol levels [142]. Obesity can increase chronic stress, in which glucocorticoid secretion from the adrenal cortex is increased [143]. In addition, the social stigma related to obesity also increases stress and long-term blood cortisol levels in humans [144]. The cortisol levels measured in hair correlates with BMI and the waist–hip ratio [145,146,147,148,149,150]. An increment of 9.8% in hair cortisol levels is associated with a 2.5 kg/m^2^ higher BMI [147]. Increased hair cortisol levels are also associated with a higher prevalence of metabolic syndrome [151].

Obesity increases 11β-HSD1 expression in visceral adipose tissue, which leads to higher local glucocorticoid availability in the tissue [152]. Abnormally high 11β-HSD1 expression is a key factor in the pathogenesis of abdominal obesity and metabolic syndrome [132]. Elevated local cortisol in adipose tissue can contribute to insulin resistance [153]. Contrary to these reports, some studies have found that 11β-HSD1 activity decreases with an increase in BMI in subjects with obesity [154,155,156]. The explanation for this discrepancy is not clear. Obesity may not always be correlated with a high blood concentration of glucocorticoids or local glucocorticoid availability.

## 13. Effects of Obesity on CX3CL1 Expression

As obesity, at least in part, increases the circulating glucocorticoids and 11β-HSD1 expression in visceral adipose tissue, the increased expression of CX3CL1 in the tissue can be expected. Obese individuals have higher CX3CL1 levels in subcutaneous adipose tissue compared with participants who are lean [157]. Plasma CX3CL1 levels were found to be increased in patients with type 2 diabetes mellitus compared with participants without this condition [157]. Consistent with these findings in humans, a high-fat diet of 20 weeks increases CX3CL1 expression in the visceral adipose tissue of mice [158]. However, administering a high-fat diet for 8 weeks decreases CX3CL1 expression in the visceral adipose tissue of 8-, 15-, and 20-week-old mice. Obese (*ob*/*ob*) mice are leptin-deficient, and are used as models for type 2 diabetes and obesity. In 8-week-old *ob*/*ob* mice, CX3CL1 expression in visceral adipose tissue is approximately 25% that of their wild-type littermates [159]. These results suggest that, while short-term feeding of a high-fat diet decreases the adipose CX3CL1 expression, a long-term high-fat diet augments it.

The CX3CL1 signaling deficiency in mice with a short-term high-fat diet resulted in reduced M2-polarized macrophage migration and an M1-dominant shift in macrophages within the visceral adipose tissue of obese mice [159]. This indicates that adipose CX3CL1 contributes to the total adipose tissue macrophage content and the ratio of M1 to M2 adipose tissue macrophages. On the contrary, CX3CL1 augmented with 20 weeks of a high-fat diet does not contribute to the total adipose tissue macrophage content, the ratio of M1 to M2 adipose tissue macrophages, the expression of inflammatory markers, and the content of T-cells [158]. The reason why CX3CL1 induced with a long-term high-fat diet does not affect tissue-resident immune cells is unknown. Considering that CX3CL1 in the peritoneal cavity signals to the hippocampus through the modulation of peritoneal immune cells [128], it could be speculated that the increased adipose CX3CL1 in obesity does not promote the hippocampus BDNF. Of note, both adipose tissue-resident macrophages and peritoneal macrophages are derived from the same origin, erythro–myeloid progenitor [160]. In this case, regardless of feeding duration, the adipose CX3CL1-mediated promotion of the hippocampus BDNF is inhibited under obese conditions induced by a high-fat diet. While aging and a short-term high-fat diet decreases the expression of CX3CL1 in visceral adipose tissue, a long-term high-fat diet inhibits the effects of adipose CX3CL1 on immune cells. Dysregulation of adipose CX3CL1 may contribute to decreased BDNF levels in the hippocampus with obesity.

## 14. Exercise Induces Glucocorticoid Secretion and Accelerates Its Termination Process

Aerobic exercise is a type of stressor that can modify the circulating levels of stress biomarkers, including glucocorticoids [161]. Acute bouts of aerobic exercise transiently elevate circulating cortisol levels [162,163]. The degree of increase in circulating cortisol is influenced by the intensity, duration, and training status of the exercise [162,164,165]. Regular aerobic exercise is associated with faster recovery from glucocorticoid secretion, which occurs through the increased conversion of cortisol into hormonally inactive cortisone. This physiological change may result in a reduced response to physical or psychological stressors [163,166,167] and appears to protect athletes from the deleterious effects of prolonged elevated cortisol secretion [161,162,168].

## 15. Exercise and CX3CL1

Exercise increases the expression level of CX3CL1 in skeletal muscles [169,170,171]. In human muscles, the expression of 938 genes was found to be changed after acute exercise [169]. Among those genes, 29 genes encode putative secreted proteins. The CX3CL1 gene is one of them, and its increase was confirmed with an ELISA assay [169].

The effects of exercise on adipose CX3CL1 expression are still unknown; however, considering the transient increase in circulating glucocorticoids after exercise, adipose CX3CL1 expression could be increased with exercise. In white adipose tissue, 11β-HSD1, which catalyzes cortisone into cortisol [130], is expressed in young mice [75]. Therefore, although exercise-induced stress is followed by an increased conversion of cortisol into hormonally inactive cortisone [161,162,168], glucocorticoid availability in white adipose tissue should be maintained by 11β-HSD1, as should the expression of CX3CL1 in the tissue. Adipose CX3CL1 may contribute to the exercise-induced augmentation of BDNF expression.

## 16. Fasting Modulates the Circadian Rhythm of Glucocorticoid Secretion

The effects of fasting on cortisol could vary with the amount and type of fasting. A 5-day fasting period produces 1.8 times more endogenous 24-h cortisol [172]. Intermittent fasting practiced during Ramadan, however, causes a significant drop in morning cortisol levels [173]. Moreover, the impact of fasting on cortisol can depend on sex and BMI. For instance, fasting tends to increase cortisol more dramatically in men and in obese/overweight individuals [174].

Fasting for 24 h delays the corticosterone peak by 2 h in rats; however, the peak magnitude is not altered [175]. Several time-restricted feeding studies have also reported a shift in the corticosterone peak to the time when feeding started [176,177]. In humans, an increase in circulating cortisol levels is seen immediately after fasting is commenced [178]. Fasting for 5 days increases cortisol levels and shifts the peak from the morning to the afternoon [172]. Fasting for 2.5 to 6 days dramatically elevates plasma cortisol levels [179,180,181]. Early time-restricted feeding (feeding between 8:00 AM and 2:00 PM) for 4 days slightly increases serum levels of cortisol in the morning [182]. These results indicate that intermittent fasting increases the level and frequency of cortisol secretion.

## 17. Fasting and CX3CL1

The expression of CX3CL1 and its receptor CX3CR1 in the hypothalamus decreased after 48 h of fasting and recovered after 4 h of re-feeding [183]. Similarly, the BDNF mRNA levels in the hypothalamus decreased after 48 h of fasting and recovered after 4 h of re-feeding. The intracerebroventricular administration of CX3CL1 to normal mice increases the level of hypothalamic BDNF mRNA. Reciprocally, CX3CR1 knockout mice show a lower expression of hypothalamic BDNF compared to their wild-type littermates. These results indicate that CX3CL1 increases the level of BDNF mRNA in the hypothalamus. However, the effects of fasting on CX3CL1 expression in adipose tissue are unknown. It has not been examined whether fasting induces CX3CL1 expression in the visceral adipose tissue.

## 18. Conclusions

Aging results in BDNF deficiency, inducing cognitive decline and increasing the vulnerability of middle-aged adults to obesity. Reciprocally, obesity accelerates cognitive aging via the dysregulation of BDNF. Visceral fat, which is responsible for the adverse health effects of obesity, tends to be increased with an advancing age. In contrast to the adverse effects of visceral adipose tissue in aged humans and animals and those with obesity, the tissues in young and non-obese mice express CX3CL1 to signal to the hippocampus, thereby maintaining the BDNF protein level. The augmentation in the hippocampus BDNF by adipose CX3CL1 is actively working in young individuals, but it is reduced in aged individuals and in obesity conditions. This mechanism can be reactivated with exercise or the repeated administration of CX3CL1 in aged mice. Table 1 summarizes the effects of aging, obesity, exercise, and fasting on the adipose CX3CL1-mediated mechanism for maintaining the hippocampus BDNF. Further research is required to elucidate the precise role of the adipose CX3CL1-mediated promotion of the hippocampus BDNF protein levels. This might provide new therapeutic targets for preventing age-associated obesity and decline in cognitive functions.

## Figures and Tables

**Table 1:** Effects of aging, obesity, exercise and fasting.

	Aging	Obesity	Exercise	Fasting
Mass of visceral adipose tissue	**↑**	**↑**	**↓**	**↓**
Cognition	**↓**	**↓**	**↑**	**↑** *^5^
Circulating glucocorticoids	± *^1^	**↑**	**↑** *^3^	**↑**
11β-HSD1 in visceral adipose tissue	**↓**	**↑**	unknown	unknown
CX3CL1 in visceral adipose tissue	**↓**	**↑** *^2^	**↑**(?) *^4^	unknown
BDNF in the hippocampus	**↓**	**↓**	**↑**	**↑**

*^1^ With advancing age, circulating glucocorticoids are slightly increased, and their diurnal amplitude flattens [184,185]. *^2^ CX3CL1 in obesity does not affect the properties of tissue-resident immune cells [158]. *^3^ Although exercise increases the circulating glucocorticoid levels, prolonged engagement in aerobic exercise results in faster recovery [161,162,163,166,167,168]. *^4^ CX3CL1 in the visceral adipose tissue is expected to be increased by exercise. The details are described in the main text. *^5^ The effects of fasting on cognitive functions are inconclusive. ↑, increase; ↓, decrease.

## Data Availability

Not applicable.
